# Non-invasive ventilation in patients with a clinical diagnosis of pneumonia: a clinical audit

**DOI:** 10.1186/cc12081

**Published:** 2013-03-19

**Authors:** CJ Wright, J Hornsby, DA Barr, SR Moonesinghe

**Affiliations:** 1 Glasgow Royal Infirmary, Glasgow, UK; 2 University College Hospital, London, UK; 3 Brownlee Institute, Glasgow, UK

## Introduction

The benefits of non-invasive ventilation (NIV) in patients with type 2 respiratory failure secondary to exacerbations of COPD are widely acknowledged [1], but its efficacy in respiratory failure of other aetiologies is less clear. We describe use of NIV (bilevel and/ or continuous positive airway pressure modes) in the subgroup of patients with a clinical diagnosis of pneumonia in a 35-bed adult critical care unit (ACCU).

## Methods

A retrospective review of data recorded prospectively on an electronic clinical information management system at University College Hospital (UCH), London, UK. Patients requiring NIV acutely at UCH are managed on the ACCU. Electronic records reviewed for patients admitted from 1 November 2010 to 2 December 2011 with clinical diagnosis of pneumonia, and in whom NIV was recorded on ≥6 occasions on the electronic chart. We concede some patients on NIV <6 hours may have been missed. We excluded patients in whom NIV was the ceiling of therapy and those using domiciliary NIV. The following data were collected: baseline patient demographics, comorbidities, therapy received in the ACCU, respiratory physiological variables, length of stay/unit outcome. Descriptive statistics of the cohort and variables associated on univariate analysis with requirement for intubation were generated using MedCalc Version 12.3.0.0 (MedCalc Software, Mariakerke, Belgium).

## Results

Fifty-two patients fulfilled the inclusion criteria, 29 (55.8%) were male, median age was 62.4 (IQR 48.8 to 71.6), median unit stay was 8 days (IQR 4.5 to 16.5), mean duration of NIV use was 2 days (IQR 1 to 3), 27 (51.9%) required intubation of whom 48% died. Total mortality was 13 (25%) - all intubated. Patients with recorded comorbidities were more likely to be intubated than those without (OR = 4.3, *P *= 0.0362); pH was significantly lower in those requiring intubation at all recorded time points; and mean FiO2 at 4 to 6 hours was significantly higher in those requiring intubation(0.72 vs. 0.56, *P *= 0.032). There was a trend toward patients with COPD requiring intubation more often (OR = 8.4, *P *= 0.0711).

## Conclusion

NIV was successful in 48.1% of patients with pneumonia, the remainder requiring intubation. Given the high mortality in those patients who failed NIV we believe its use in a ward setting is hazardous. We conclude that NIV may be useful in some individuals with pneumonia, but they should be managed in the ACCU setting. Further work is required to identify those patients at risk of deterioration on NIV given the possible excess mortality.
